# Low dose aspirin and cognitive function in middle aged to elderly adults: randomised controlled trial 

**DOI:** 10.1136/bmj.a1198

**Published:** 2008-09-01

**Authors:** Jackie F Price, Marlene C Stewart, Ian J Deary, Gordon D Murray, Peter Sandercock, Isabella Butcher, F Gerald R Fowkes

**Affiliations:** 1 Division of Community Health Sciences and Centre for Public Health and Primary Care Research, University of Edinburgh, Edinburgh; 2 Psychology in the School of Philosophy, Psychology and Language, University of Edinburgh; 3 Department of Clinical Neurosciences, University of Edinburgh

## Abstract

**Objective** To determine the effects of low dose aspirin on cognitive function in middle aged to elderly men and women at moderately increased cardiovascular risk.

**Design** Randomised double blind placebo controlled trial.

**Setting** Central Scotland.

**Participants** 3350 men and women aged over 50 participating in the aspirin for asymptomatic atherosclerosis trial.

**Intervention** Low dose aspirin (100 mg daily) or placebo for five years.

**Main outcome measures** Tests of memory, executive function, non-verbal reasoning, mental flexibility, and information processing five years after randomisation, with scores used to create a summary cognitive score (general factor).

**Results** At baseline, mean vocabulary scores (an indicator of previous cognitive ability) were similar in the aspirin (30.9, SD 4.7) and placebo (31.1, SD 4.7) groups. In the primary intention to treat analysis, there was no significant difference at follow-up between the groups in the proportion achieving over the median general factor cognitive score (32.7% and 34.8% respectively, odds ratio 0.91, 95% confidence interval 0.79 to 1.05, P=0.20) or in mean scores on the individual cognitive tests. There were also no significant differences in change in cognitive ability over the five years in a subset of 504 who underwent detailed cognitive testing at baseline.

**Conclusion** Low dose aspirin does not affect cognitive function in middle aged to elderly people at increased cardiovascular risk.

**Trial registration** ISRCTN 66587262.

## Introduction

Cognitive function declines with age, and such decline is of increasing public health concern because of the burden it imposes on individuals, carers, and the wider society.^1^ ^2^ Reduced levels of cognitive performance in older age have been associated with the presence of cardiovascular disease,^3^ ^4^ possibly mediated through multiple small cerebral infarcts from microatheroma or thromboses in the brain. Antithrombotic medication such as aspirin might therefore have a role in the preservation of cognitive function, particularly in individuals at increased risk of atherosclerotic vascular disease. Conversely, if aspirin promotes microhaemorrhages in the brain, this might exacerbate cognitive decline. Observational studies have reported either no association between regular use of aspirin or other non-steroidal anti-inflammatory drugs and cognitive decline in older age^5^ ^6^ or a modest trend towards protection of cognitive function.^7^ ^8^ ^9^ ^10^ Data from randomised controlled trials, however, are sparse. The results of a recent large clinical trial indicated that long term low dose aspirin had no effect on memory in healthy women aged over 64, with inconclusive findings for executive function.^11^ Conversely, a much smaller trial in men aged over 55 without dementia who were at risk of cardiovascular disease suggested a beneficial effect of low dose aspirin on verbal fluency and mental flexibility.^12^

We determined the effects of long term low dose aspirin on a wide range of cognitive functions that are known to decline with age in a relatively young population (aged 50 and over) of men and women at moderately high vascular risk. We included two cognitive assessments, one at baseline and one after five years, in an ongoing randomised controlled trial of daily aspirin (100 mg enteric coated) in people aged over 50, in which the primary end points were cardiovascular events and death (the aspirin for asymptomatic atherosclerosis (AAA) trial). At randomisation, all participants in this trial were at moderately increased cardiovascular risk because of a low ankle brachial index, a reduced ratio of systolic blood pressure in the ankle to that in the arm, which is a good predictor of increased mortality and cardiovascular events.^13^

## Methods

### Participants

This double blind, placebo controlled randomised clinical trial took place in central Scotland (Lanarkshire, Edinburgh, and Glasgow) in 1998-2006. All participants gave written informed consent. Volunteers aged 50-75 were recruited by direct mailing of people registered with participating general practices (83% of practices contacted agreed to participate). People were ineligible for screening if they had had a previous myocardial infarction or stroke or if they were taking aspirin or warfarin. The study was advertised in the local press and by posters in general practices.

At a screening clinic researchers recorded right and left brachial, posterior tibial, and dorsalis pedis systolic pressures with a standard sphygmomanometer and a Doppler probe. The ankle brachial index was calculated as the ratio of the lowest pressure in either ankle to the higher of the measurements in the left or right arm. Exclusion criteria instituted at the screening clinic included an ankle brachial index >0.95 in both legs; current use of aspirin, other antiplatelet, or anticoagulant; severe indigestion; history of myocardial infarction, stroke, angina, or peripheral arterial disease; chronic liver or kidney disease; chemotherapy; contraindications to treatment with aspirin; and an abnormally high or low packed cell volume.

Participants were randomised to either daily aspirin (100 mg enteric coated) or identical placebo (both preparations provided by Bayer HealthCare). Consecutive subject study numbers were assigned to aspirin or placebo with permuted blocks of size eight, which varied randomly. A specialist member of staff who was not otherwise involved in the study produced the randomisation list. Staff at a single dedicated pharmacy labelled bottles containing aspirin or placebo with the appropriate subject numbers. Recruits entering the trial were then allocated the next available study number and provided with the appropriately labelled bottle containing one year’s supply of tablets. Randomisation lists in sealed envelopes were available only to the pharmacy, to an independent member of staff who was responsible for unblinding individual participants in the case of clinical necessity, and to an independent statistician responsible for providing annual reports to the data monitoring committee. Staff working on the trial, the investigators, and participants remained blinded to treatment allocation.

Participants were contacted annually by telephone and newsletter, in between which they were encouraged to report admission to hospital or cessation of study medication. If they stopped study medication other than for a medical reason, they were actively encouraged to start again. If they started taking aspirin or another antiplatelet drug, their study medication was discontinued. Participants recorded their compliance in a diary. A specially trained research nurse assessed self reported compliance annually, after which supplies of study tablets were renewed by post.

### Cognitive function testing

Participants underwent neuropsychological tests three months after they had started the trial medication (baseline cognitive testing) and at five years. At baseline, they completed a combined version of the Junior and Senior Form A synonyms of the Mill Hill vocabulary scale.^14^ They were presented with a word and asked to identify the closest synonym from six given alternatives. The Mill Hill scale, in common with other vocabulary tests, is primarily an indicator of previous (“best ever” or premorbid) cognitive ability, which changes little with age.^15^ ^16^ It was used to compare the baseline characteristics of the trial population and to enable assessment of the impact of any difference in previous cognitive ability on loss to follow-up and on change in cognitive performance over time. The test has been shown previously to correlate highly with other tests of previous cognitive ability that are used in a similar manner.^17^

At about five years, participants underwent a battery of tests in a predetermined order to assess a broad range of cognitive functions (230 participants were tested after four years, 259 after six years, two after seven years, and the remainder at five years). The mini-mental state examination^18^ was included as a general mental assessment and is often used as a “screen” for dementia. Executive function was assessed with the verbal fluency test,^19^ which requires participants to generate as many words as possible with a specified initial letter (C, F, and L). As a measure of non-verbal reasoning, participants worked through all five sets (A to E, containing 12 items each) of Raven’s progressive matrices^14^ and were scored according to the number of items they completed correctly within 20 minutes. Immediate and delayed memory was assessed with the total score on the first five trials (I through V, constituting the same list of 15 words) of the auditory verbal learning test.^19^ The trail making test^20^ measured mental flexibility, and the time taken to complete part B was used in the subsequent analysis. In the digit symbol test,^21^ used as a measure of speed of information processing, we recorded the number of symbols matched correctly to their corresponding numbers in 90 seconds. The hospital anxiety and depression scale (A and D)^22^ was also used for the assessment of mood states, as these can affect performance on the tests. At follow-up the national adult reading test measured premorbid cognitive ability.^23^

In addition, we undertook a highly sensitive assessment of cognitive change over time in a “cognitive change subset” of recruits who underwent the same detailed battery of tests as above at baseline. We recruited 504 participants at baseline for this purpose from consecutive participants who agreed to complete the Mill Hill vocabulary scale during the period May 2000 to March 2001. Baseline and follow-up cognitive test scores were available on 399 participants in this subset.

### Data analysis

We used each participant’s score on the Carstairs deprivation index, based on the postal code classification from the 1991 census,^24^ ^25^ to assign a deprivation category ranging from I (most affluent) to VII (most deprived).^24^ Further statistical analysis was carried out with SAS/STAT software, version 9.1, of the SAS System for Windows (SAS Institute, Cary, NC).

Our primary analysis was on an intention to treat basis, using both individual test scores and the proportion of participants achieving over an aggregate cognitive score. For the latter, those not achieving the aggregate score would include those failing to complete cognitive testing at follow-up, thereby reducing bias from death or unwillingness to complete the tests (likely to be associated with greater cognitive decline). We estimated that 3300 participants would give 93% power at 5% significance to detect a difference of 47% versus 53% attaining the threshold. For the cognitive change subset, we estimated that two groups of 200 would provide 80% power to detect a treatment effect size of 0.28 standard deviations at 5% significance (90% power for 0.33 SD). Allowing for loss to follow up, we aimed to recruit at least 480 participants into the subset at baseline.

Principal components analysis was carried out to determine a summary cognitive score (the general factor) for the cognitive function variables (auditory verbal learning, Raven’s matrix, digit symbol, verbal fluency, and trail making) with scree slope analysis to determine the number of factors. Where one or two of the test results were missing for an individual, we performed multiple imputation, accounting for age, reading score, sex, and deprivation score. This analysis resulted in a single component (the general factor, reflecting general cognitive ability^26^), which explained 58% of the total variance (standardised scoring coefficients: auditory verbal learning 0.23, Raven’s matrices 0.28, digit symbol 0.29, verbal fluency 0.22, trail making −0.28). This component was extracted as the first unrotated principal component. We subsequently used the median general factor score for all participants with such a score available as the cut-off point for the aggregate cognitive score used to assign participants into two groups in the primary analysis.

We determined differences in mean cognitive test scores between the aspirin and placebo groups at follow-up using Student’s *t* tests. For the cognitive change subset, results are presented as estimates (with 95% confidence intervals) of the difference between the two treatments at follow-up for the factor score and for each individual test of cognition. The difference between the treatments for each variable was estimated after adjustment for baseline values in the first model and baseline values, age, sex, ankle brachial index, total plasma cholesterol concentration, smoking status, and deprivation category in the second model.

Interim analyses of cognitive data were not conducted during the course of the study. The primary end points, including the aggregate cognitive score, and statistical comparisons were prespecified. All P values were two sided and were not adjusted for multiple testing.

## Results

### Primary analyses

Of the 28 980 participants screened, 24 066 were excluded because they had an ankle brachial index >0.95 in both legs. Before randomisation, 631 did not meet other eligibility criteria and 933 declined to take part (figure). The 3350 remaining were randomly assigned to aspirin or placebo. Table 1 shows the characteristics of the two groups at randomisation.[Table tbl1] In total, 2733 (81.6%) participants (1361 in the aspirin group and 1372 in the placebo group) agreed to complete the Mill Hill vocabulary scale (MHVS). Mean scores on this test were similar between the two groups (30.9 (SD 4.7) and 31.1 (SD 4.7), respectively).

**Figure fig1:**
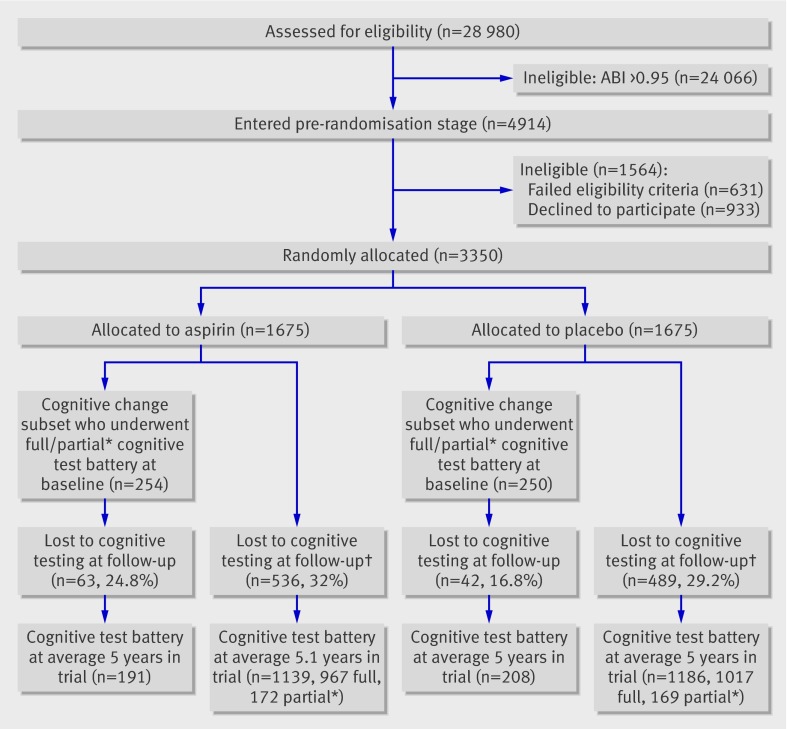
Study population and follow-up. *At least one cognitive test in battery of tests completed. †Breakdown of reasons for loss to follow-up by treatment allocation not given to preserve blinding within main trial until cardiovascular end point reached. See text for breakdown of reasons for total trial population (including 160 deaths)

**Table 1 tbl1:** Characteristics of participants* at randomisation. Figures are means (SD) unless stated otherwise

Characteristic	Aspirin group (n=1675)	Placebo group (n=1675)
Age (years)	62 (7)	62 (7)
No (%) of men	481 (29)	473 (28)
No (%) by Carstairs deprivation category†:		
I/II	287 (17)	300 (18)
III/IV/V	927 (55)	884 (53)
VI/VII	458 (27)	489 (29)
Ankle brachial index	0.86 (0.09)	0.86 (0.09)
Systolic blood pressure (mm Hg)	149 (22)	147 (22)
Diastolic blood pressure (mm Hg)	84 (11)	84 (11)
Total plasma cholesterol (mmol/l)	6.2 (1.1)	6.2 (1.1)
No (%) of smokers:		
Current	548 (33)	539 (32)
Previous‡	540 (32)	561 (34)
Never	587 (35)	575 (34)
Mill Hill vocabulary scale score	30.9 (4.7)	31.1 (4.7)
No (%) by recruitment centre:		
Edinburgh	388 (23)	364 (22)
Glasgow	478 (29)	497 (30)
Lanarkshire	809 (48)	814 (48)

*Data available for all participants except for deprivation score (n=1673 in both groups), diastolic blood pressure (n=1674 in aspirin group and 1671 in placebo group), plasma cholesterol (n=1663 and 1666), Mill Hill vocabulary scale score (n=1361 and 1372).

†I (most affluent) to VII (most deprived).

‡Smokers who had stopped smoking for at least 6 months before randomisation.

A total of 1025 participants were lost to cognitive follow-up: 317 withdrew (n=157) or died (n=160) before testing, 668 refused the cognitive assessments either in a research clinic or in their own homes, 27 could not be contacted, and 13 were not tested for practical reasons (including serious illness and living abroad). Table 2 shows the characteristics at randomisation of the participants who completed at least one cognitive test at follow-up compared with those not cognitively tested.[Table tbl2] Those not tested were slightly older, more likely to be men, and more socially deprived, with a poorer vascular risk factor profile. Differences between the tested and untested groups in previous cognitive ability were small (vocabulary score 31.1 (SD 4.7) *v* 30.6 (SD 4.6), P=0.03).

**Table 2 tbl2:** Characteristics of participants* at randomisation by cognitive test status at follow-up. Figures are means (SD) unless stated otherwise

Characteristic	Cognitively tested (n=2325)	Not cognitively tested (n=1025)	P value†
Age (years)	62 (7)	63 (7)	0.001
No (%) of men	627 (27)	327 (32)	0.004
No (%) by Carstairs deprivation category‡:			
I/II	444 (19)	143 (14)	<0.001
III/IV/V	1269 (55)	542 (53)	
VI/VII	610 (26)	337 (33)	
Ankle brachial index	0.86 (0.09	0.85 (0.10	0.003
Systolic blood pressure (mm Hg)	147 (21)	149 (22)	0.02
Diastolic blood pressure (mm Hg)	84 (11)	83 (11)	0.26
Total plasma cholesterol (mmol/l)	6.2 (1.1)	6.2 (1.1)	0.99
No (%) of smokers:			
Current	671 (29)	416 (41)	<0.001
Previous§	779 (33)	322 (31)	
Never	875 (38)	287 (28)	
Mill Hill vocabulary scale score	31.1 (4.7)	30.6 (4.6)	0.03

*Data available for all participants except for deprivation category (n=2323 in tested group, 1022 in untested group), diastolic blood pressure (n=2322 and 1023), plasma cholesterol (n=2314 and 1015), Mill Hill vocabulary scale score (n=2202 and 531).

†P values from χ^2^ test for categorical variables and *t* test for continuous variables except ankle brachial index (Mann-Whitney test).

‡I (most affluent) to VII (most deprived).

§Smokers who had stopped smoking for at least 6 months before randomisation.

Of the 2325 participants cognitively tested, 1984 completed the full test battery. Fourteen omitted the adult reading test only and 264 omitted only one or two of the tests used to generate the general cognitive factor score (in which case we calculated their general factor score using imputation). Thus a general factor score was calculated for 2262 participants (1109 in aspirin group and 1153 in placebo group). For the purposes of the primary end point analysis, all 1088 participants without a general factor score at follow-up were included in the group who did not achieve the aggregate score.

Overall, 32.7% (n=548) of participants in the aspirin group and 34.8% (n=583) in the placebo group achieved over the median general factor score (odds ratio 0.91, 95% confidence interval 0.79 to 1.05, P=0.20). There was little change in the odds ratio after adjustment for age, sex, ankle brachial index, deprivation category, smoking status, and total plasma cholesterol (0.93, 0.80 to 1.08, P=0.35). These results did not alter greatly when we repeated analyses excluding participants without a general factor score, with proportions of 49.4% and 50.6%, respectively, achieving over the median score (unadjusted 0.96, 0.81 to 1.13, P=0.59; adjusted 0.98, 0.82 to 1.17, P=0.83).

Table 3 shows the mean test scores at follow-up in the two groups.[Table tbl3] There was no significant difference between the two groups for any of the individual tests or for the general factor. The proportion of participants scoring below 24 on the mini-mental state examination was similar (2.4% (27) *v* 2.5% (29), P=0.91). There was also no significant difference in mean scores on the hospital anxiety scale (5.2 (SD 3.6) *v* 5.4 (SD 3.6), P=0.12) or the hospital depression scale (2.9 (SD 2.5) *v* 3.0 (SD 2.7), P=0.82).

**Table 3 tbl3:** Performance on cognitive function tests at follow-up*

Test of cognition	Aspirin group (n=1139)			Placebo group (n=1186)		P value
	No of participants	Mean (SD) score; 95% CI		No of participants	Mean (SD) score; 95% CI	
General cognitive factor score (summary cognitive score)†	1109	0.00 (1.01); −0.06 to 0.06		1153	−0.01 (0.99); −0.06 to 0.05	0.83
Raven’s progressive matrices (5 sets of 12 item tests; maximum possible score 60)	1110	34.3 (9.5); 33.8 to 34.9		1153	34.4 (9.3); 33.9 to 35.0	0.83
Auditory verbal learning, trials I-V (sum of five trials with same list; maximum possible 75 words)	1118	63.0 (16.7); 62.1 to 64.0		1159	63.0 (16.9); 62.0 to 64.0	0.93
Digit symbol (total No of symbols matched correctly in 90 second test; maximum possible score 93)	1126	40.0 (11.7); 39.3 to 40.7		1170	40.0 (11.7); 39.4 to 40.7	0.92
Verbal fluency (total No of words generated in three 1 minute tests)	1117	37.6 (12.8); 36.9 to 38.4		1156	37.1 (12.7); 36.3 to 37.8	0.27
Trail making (seconds to completion)‡	1122	4.6 (0.4); 4.6 to 4.6		1167	4.6 (0.4); 4.6 to 4.6	0.90
Mini-mental state examination (total score, maximum possible 30)	1131	28.6 (1.7); 28.5 to 28.7		1178	28.5 (1.8); 28.4 to 28.6	0.20

*In all tests except trail making, higher scores indicate better function. Imputed test scores were used for individuals with ≤2 scores missing for Raven’s progressive matrices (162 scores imputed), auditory verbal learning (95), digit symbol (10), verbal fluency (45), and trail making (37).

†Calculated from factor analysis with unrotated principal component analysis with current cognitive function measures excluding mini-mental state.

‡Natural log of time in seconds to complete trail making test (untransformed means 102 seconds for aspirin group and 101 seconds for placebo group).

### Analysis of cognitive change subset

Table 4 shows that there were no significant differences in the change in cognitive ability over the five years for any of the individual tests or for the general factor between the treatment groups. Scores for tests of speed of processing information and mental flexibility in the cognitive change subset declined over the five years, though the change in mental flexibility scores was not significant (mean digit symbol 45.0 at baseline, 42.4 at follow-up, P<0.001: mean trail making 94.2 seconds at baseline, 97.4 seconds at follow-up, P=0.2). There was no significant difference over time in verbal fluency or non-verbal reasoning and scores on the memory test improved (mean auditory verbal learning 63.8 at baseline, 68.5 at follow-up, P<0.001). Changes observed over time are probably underestimates of the true age related declines^26^ because the effects of age can be ameliorated by familiarity with the test.

**Table 4 tbl4:** Change in mean (SD) cognitive test scores in cognitive change subset participating at baseline and follow-up*

Test of cognition†	Aspirin			Placebo			Difference (aspirin-placebo) (95% CI), P value	
	Follow-up score	Change from baseline‡		Follow-up score	Change from baseline‡		Adjusted for baseline value	Fully adjusted§
General factor score	0.39 (0.89)	0.26 (0.45)		0.37 (0.89)	0.27 (0.47)		−0.01 (−0.09 to 0.08), 0.89	0.01 (−0.07 to 0.09), 0.77
Raven’s progressive matrices	37.8 (8.9)	−0.1 (5.9)		37.5 (8.9)	−0.6 (5.4)		0.5 (−0.6 to 1.6), 0.34	0.7 (−0.3 to 1.8), 0.17
Auditory verbal learning	68.0 (15.2)	3.9 (11.5)		68.3 (16.1)	5.2 (11.2)		−1.1 (−3.3 to 1.1), 0.32	−0.7 (−2.9 to 1.4), 0.51
Digit symbol	42.7 (10.8)	−2.6 (6.2)		42.3 (11.6)	−2.5 (6.3)		0.0 (-1.2 to 1.2), 0.96	0.3 (−0.9 to 1.5), 0.62
Verbal fluency	41.4 (11.5)	0.6 (7.5)		41.3 (11.3)	0.8 (7.8)		−0.1 (−1.6 to 1.3), 0.88	−0.2 (−1.6 to 1.3), 0.84
Trail making	4.50 (0.37)	0.02 (0.28)		4.51 (0.38)	0.02 (0.31)		0.00 (−0.06 to 0.06), 1.00	−0.01 (−0.07 to 0.04), 0.61

*No of participants for each analysis is 399 (including 25 with some imputation at follow-up), 191 in aspirin group and 208 in placebo group.

†See table 3 for details of tests.

‡Score at follow-up minus score at baseline.

§Additional adjustment for age, sex, and baseline values for ankle brachial index, total plasma cholesterol, smoking status, and Carstairs deprivation category.

Interaction terms for test specific baseline covariate and treatment group were examined and none was significant.

To examine whether compliance might have affected our results, we performed an on treatment analysis for participants who reported that they had taken their study medication for at least two thirds of the year before follow-up. This analysis was planned before unblinding the treatment allocations, but after the data had been collected. Some 1708 participants were confirmed as still taking treatment. Of the 1642 remaining, 160 had died, 269 had had a cardiovascular event, 203 had started taking aspirin for a reason other than a cardiovascular event, 317 had reported known side effects of aspirin or had started medication contraindicated with aspirin, 250 had developed other symptoms or illnesses unrelated to aspirin, and 404 simply no longer wanted to take the study medication (status of 27 was unknown and 12 were still taking study medication but for less than two thirds of preceding year).

Of the participants still taking treatment at follow-up, 850 were in the aspirin group and 858 were in the placebo group. There were no significant differences in baseline characteristics between these two groups (data not shown). In each group 42.8% (364) and 45.8% (393) achieved over the median general factor score (0.89, 0.73 to 1.07, P=0.22). Of the 2325 participants cognitively tested at follow-up, 1505 (64.7%) were still taking treatment (743 and 762, respectively). The characteristics at randomisation of these two groups were also similar (data not shown). Mean (SD) cognitive scores at follow-up for the aspirin and placebo treatment groups, respectively, were general factor 0.0 (1.0) in both groups; Raven’s matrix 34.3 (9.6) and 34.8 (9.2); auditory verbal 63.5 (16.7) and 63.3 (16.7); digit symbol 40.6 (11.9) and 40.8 (11.7); verbal fluency 37.8 (13.1) and 37.8 (12.7); trail making 4.6 (0.4) and 4.6 (0.4); and mini-mental state <24 2.7% (n=20) and 1.9% (n=14, P=0.26).

## Discussion

In this large double blind, placebo controlled randomised clinical trial we found no significant difference in measures of cognitive function between people randomised to 100 mg aspirin daily compared with placebo over a five year period. Our initial hypothesis was based on observed associations between cardiovascular disease and cognitive decline^3^ ^4^ and the positive effects of aspirin on the incidence of cardiovascular events in both primary and secondary prevention trials,^27^ ^28^ although it has been argued that aspirin might alter the presentation of cardiovascular disease rather than affecting underlying disease processes.^29^ ^30^ There is also evidence that non-steroidal anti-inflammatory drugs might reduce neurotoxic inflammatory responses in the brain or reduce the cellular response to excessive stimulation by excitotoxins, or both.^10^ Our results, however, do not support a beneficial effect of aspirin on cognitive performance in middle aged to elderly people at increased cardiovascular risk. While aspirin could be expected to have a beneficial role in reducing large vessel cerebral occlusive disease and small vessel disease caused by “microatheroma,” it might not influence (and might even exacerbate) other forms of small vessel disease, such as arteriosclerosis with diffuse white matter damage from ischaemia or “leaky vessels,”^31^ with or without microhaemorrhages.

### Veracity of findings

We studied men and women at increased risk of developing symptomatic cardiovascular disease in whom the effect of aspirin might be expected to be accentuated (correlations of between 0.03 and 0.15 between ankle brachial index and cognitive function have been reported, depending on the cognitive tests performed^32^). The trial had sufficient power to detect small differences in cognitive function between the groups, and we used various tests to assess a range of cognitive abilities. As virtually all neuropsychological tests are highly intercorrelated, we used a summary cognitive score that represents their shared variance to determine the effect of aspirin on global cognitive capacity. As each test measures a specific aspect of cognition that might differ in its response to treatment we also analysed the test scores individually. In our robust statistical analyses we used a prespecified primary outcome measure that took into account possible associations between performance on the cognitive tests and ability or willingness to complete the cognitive test at follow-up.

The effect of aspirin on cognitive performance might be more evident in younger people,^7^ and cerebral damage caused by micro-thromboses and other neurodegenerative processes might start to become established in middle age. This hypothesis, however, was not supported by our post hoc analyses (data not shown) in which separation of the total trial population into those aged 50-64 and those aged 65 or older had little impact on the overall results.

Inability to study the effects of aspirin on younger people was one of the limitations of a recent clinical trial by Kang et al of the effects of aspirin on memory.^11^ Another was its restriction to women. Again in post hoc analysis, we found no effect of aspirin on verbal memory in women (mean auditory verbal score 65.2 in aspirin group *v* 65.4 in placebo group, P=0.84) or men (57.2 *v* 56.5, P=0.62). Crucially, we also studied the effect of aspirin on cognitive functions other than memory, including executive function, non-verbal reasoning, mental flexibility, and information processing. Our findings that aspirin had no effect on these additional measures of cognitive ability contrast with those from the only other clinical trial of low dose aspirin to investigate similar outcomes.^12^ The latter was a relatively small trial (n=405), however, involving the cognitive testing of a subgroup of men still participating in a clinical trial several years after randomisation. Furthermore, there was no baseline measure of cognitive function, and some participants received warfarin as well as aspirin.

### Limitations and strengths

A high proportion of participants (30%) failed to complete the cognitive tests at follow-up. This level of non-testing was largely inevitable in a long term intervention study in “healthy” volunteers (the numbers included deaths and trial withdrawals as well as those who refused to take tests) and was consistent with previous studies of a similar design. In the study by Kang et al, only 71% of women initially randomised to aspirin or placebo and selected to take part in the cognitive study agreed to undergo both baseline and follow-up cognitive testing four years later.^11^ Relatively high levels of non-testing could have biased our findings, especially as it has been shown previously that people with poor cognitive function are more likely to withdraw from studies.^33^ In our study, however, there was only a small difference in Mill Hill vocabulary scores at baseline between those who did and did not undergo cognitive testing at follow-up. The potential effect of cognitive decline over the period of the study on test completion was further addressed by use of the aggregate cognitive score in our primary analysis.

Despite concerted efforts to maintain participants’ compliance with study medication, this fell throughout the duration of the trial and could have affected our results. For the purposes of the “on treatment” analysis, we used a rigorous definition of on treatment and included only participants who reported that they had taken their study medication for at least two thirds of the year before follow-up. This was consistent with definitions used in previous primary prevention trials.^34^ All participants randomised to aspirin, however, took their study medication for at least some of the trial period before stopping at various stages throughout the trial, such that the total proportion of person years on aspirin was actually much higher than that suggested by the number of participants included in the on treatment analysis. The issue of compliance was further addressed by the on treatment analysis itself, which failed to show any difference in the aggregate or mean cognitive scores between those taking aspirin and those taking placebo, or indeed any shift in the overall results that might have indicated an effect of aspirin.

We cannot exclude the possibility that higher doses or longer duration of aspirin treatment, or both, would have led to different results. The participants, although at increased risk of developing symptomatic cardiovascular disease, were free from clinical cardiovascular disease at baseline and otherwise generally healthy. Our follow-up period, although comparing favourably with previous trials on age related cognitive decline, might have been short for such a relatively young and healthy cohort. We could not assess the actual degree of cognitive decline in the population because performance on a given cognitive test (especially memory tests) is known to improve as the test is repeated, even after several years, simply because participants are familiar with the testing procedure (learning effect).^35^ ^36^ ^37^ A population with advanced vascular disease, dementia, or established mild cognitive impairment might have had a different response, as might any other population with more marked middle to late life cognitive decline. Longer follow-up into older age might also be necessary to show any “delayed” effect of aspirin from the time of altered cerebral pathology to development of observable changes in cognitive decrements.

**What is already known on this topic**

Atherosclerotic cardiovascular disease might accelerate age related cognitive decline
Aspirin has a protective role in the secondary prevention of cardiovascular disease and might also reduce age related cognitive decline

**What this study adds**

Low dose aspirin over five years did not produce any cognitive benefit in men and women aged over 50 years without dementia and at moderately increased risk of cardiovascular disease

## Notes

We thank the participants and trialists of the aspirin for asymptomatic atherosclerosis trial and staff at the Wellcome Trust clinical research facility in Edinburgh where some of the cognitive testing was performed.

Participators in the AAA Trial

Trial management and data analysis: F G R Fowkes, J F Price, G D Murray, I J Deary, M C W Stewart, A F Douglas, S Cudmore, M Bree, S A Wilson, E M Armstrong, G S McHugh, I Butcher. Data monitoring committee: S M Cobbe, C Warlow, I Ford, J C Petrie. Trial steering committee: M Bain, R Clements, J Darnborough, I Deary, F G R Fowkes, K A A Fox, G Leng, G D O Lowe, E Mallinson, G D Murray, A Pell, J F Price, A Rumley, P Sandercock, J Wrench. Outcome events committee: F G R Fowkes, J F Price, T Sommerfield, E Bream, D Northridge, R Lindley, M Bree, R Walton, C A M Ritchie. Data handling: E Healy, H Peterson, E Crooks, J Hay, E M Kerracher, N Kerracher, A Sloan, D Thom, L C McGoohan. Blood assays and pharmacy services: J Patterson, G Baxter, R Spooner, E Foley, J Carracher. Data collection: E Tolmie, E C Graham, J F Alexander, H Lawrie, E M Armstrong, I F Tierney, S A Wilson, P Ross, F Reston, D Willis, E M Kerracher, F J Neary, F B Smith, K Hepburn, C D Rea, H Mackay, W A Smith, C D Martin, C A M Ritchie, L Paton.

Ethical approval: Ethics committees of Lanarkshire, Lothian, and Greater Glasgow Health Boards.

Provenance and peer review: Not commissioned; externally peer reviewed.

## Notes

**Cite this as:** *BMJ* 2008;337:a1198
